# Effects of Personality Traits on the Severity of Chronic Subjective Tinnitus: A Cross-Sectional Analysis

**DOI:** 10.3390/medicina60081259

**Published:** 2024-08-03

**Authors:** Hyoyeon Jeong, Ikhee Kim, Seung Ho Kim, Jong Bin Lee, Hantai Kim

**Affiliations:** 1 Department of Otorhinolaryngology–Head and Neck Surgery, Konyang University College of Medicine, Daejeon 35365, Republic of Korea; dus5297@gmail.com (H.J.); kwingh7.7@gmail.com (I.K.); 400867@kyuh.ac.kr (S.H.K.); rogue25@kyuh.ac.kr (J.B.L.); 2 Department of Medicine, The Graduate School of Konyang University, Daejeon 35365, Republic of Korea

**Keywords:** tinnitus, personality, big five, Helplessness, trauma

## Abstract

*Background and Objectives*: We hypothesized that an individual’s personality traits would have an impact on the distress of subjective tinnitus. To investigate this, 32 participants were recruited; we followed up with this cohort. This study is a cross-sectional analysis of a part of this cohort, identifying how their personality traits make a difference in the severity of tinnitus distress. *Materials and Methods*: Thirty-two participants completed a personality test based on the Big Five theory, consisting of 160 items. Additionally, the severity of tinnitus was assessed using the Tinnitus Handicap Inventory (THI), and the accompanying level of depression was measured using the Beck Depression Inventory (BDI). Audiometry tests, including pure-tone audiometry, were also conducted. Participants were categorized into the ‘mild group’ if their total THI score was 36 or below, and into the ‘severe group’ if their score was 38 or above. *Results*: In the traditional five domains of the Big Five Inventory (Openness, Conscientiousness, Extraversion, Agreeableness, Neuroticism), only the ‘Neuroticism’ domain showed a difference between the two groups (25.1 ± 21.0 in the mild group and 43.1 ± 18.2 in the severe group, *p* = 0.014). Among ego-related factors, the ‘Helplessness’ domain (26.3 ± 22.9 in the mild group and 62.3 ± 27.9 in the severe group, *p* < 0.001) and the communication-related factor of ‘Listening’ (58.7 ± 18.8 in the mild group and 37.8 ± 27.9 in the severe group, *p* = 0.020) showed differences between the two groups. In the multivariate analysis, ‘Helplessness’ (estimate 0.419, 95% confidence interval 0.249–0.589, *p* < 0.001), ‘Emotional Trauma’ (0.213, 0.020–0.406, *p* = 0.032), and ‘Pure-tone threshold at 2000 Hz’ (0.944, 0.477–1.411, *p* < 0.001) were identified as factors influencing the severity of tinnitus distress. *Conclusions*: Ego-related factors, particularly Helplessness and Emotional Trauma, can influence tinnitus distress and should be considered in the management of tinnitus.

## 1. Introduction

When a person perceives a sound in their ear without any actual external source, it is called tinnitus [1,2]. While there is some regional variation, the prevalence in the United States is estimated to be around 8–25.3%; some reports suggest that as many as 1–7% of patients are severely affected [3,4]. Consequently, tinnitus is one of the most common symptoms reported by patients visiting otolaryngology clinics [5]. However, these patients are generally less satisfied with their treatment. In a survey study, nearly 80% of tinnitus patients felt that their healthcare providers did not manage their tinnitus effectively [6].

In certain specific forms of tinnitus, effective control can be achieved through medication or surgery. For instance, pulsatile tinnitus can be managed surgically by resurfacing the vascular area [7,8,9,10], and typewriter tinnitus can be treated with medications such as carbamazepine [11,12,13]. Additionally, tinnitus caused by middle ear myoclonus can be addressed through either medication or surgical intervention [14,15,16]. However, these cases are limited, and the majority of tinnitus cases are classified as chronic subjective tinnitus, which is difficult to manage and includes most patients with poorly controlled tinnitus. Various guidelines for the management of chronic subjective tinnitus emphasize the importance of education and counseling, particularly through cognitive behavioral therapy (CBT) and tinnitus retraining therapy (TRT) [17,18,19,20].

In clinical practice, TRT or CBT is often implemented based on these principles, but patient receptivity to these treatments varies. This variability leads to the hypothesis that certain personality traits might influence how patients perceive tinnitus and accept treatment modalities. While this hypothesis is not novel and has been explored in several studies [21,22,23,24,25,26,27], most of these studies have been conducted in Western cultures. Given the cultural differences in personality traits [28,29,30,31], it may be inappropriate to directly apply these findings to East Asian patients. Studies focusing on East Asian populations are relatively scarce [32,33], highlighting the need for more research on the relationship between tinnitus and personality traits in East Asian patients.

This study aims to investigate the differences in personality types according to the severity of tinnitus distress in Korean patients. We utilized the Big Five personality traits, which are widely recognized and stable measures of individual personality [34,35]. This study is distinct in that it is the first to apply the Big Five personality traits to Korean tinnitus patients.

## 2. Materials and Methods

### 2.1. Participants

Patients who had experienced chronic subjective tinnitus for more than three months and visited the Department of Otorhinolaryngology–Head and Neck Surgery, Konyang University Hospital between March 2023 and February 2024 were invited to participate in the study if they met the following criteria: (1) adults aged between 20 and 70 years, (2) an average pure-tone threshold of 45 dB or less at 500–4000 Hz, and (3) the ability to independently complete questionnaires and other assessments.

Patients diagnosed with depression, anxiety disorders, or those currently on medication for these conditions were excluded. Additionally, patients whose hearing could be improved through surgical intervention (e.g., tympanic membrane perforation) were also excluded. Participation was also restricted if high-frequency hearing loss due to noise exposure or ototoxic drugs was identified as the cause of tinnitus.

Ultimately, 32 patients were included in the study. All procedures performed in this study involving human participants were in accordance with the ethical standards of the Institutional Review Board of Konyang University Hospital (KYUH2022-09-015) and with the 1964 Helsinki Declaration and its later amendments or comparable standards. Informed consents have been obtained from all participants.

### 2.2. Audiologic Test Battery

Pure-tone audiometry (PTA) and a speech discrimination test were performed to evaluate hearing. PTA measured hearing thresholds at 500, 1000, 2000, 4000, and 8000 Hz. For speech discrimination, the examiner read 25 one-syllable words at each participant’s Most Comfortable Level (MCL) threshold, and the percentage of correctly repeated words was recorded. Tests were conducted for both ears, and the average results from both ears were used to determine each participant’s hearing level.

### 2.3. The Severity of Tinnitus and Depression Screening Questionnaire

The severity of tinnitus was measured using the Tinnitus Handicap Inventory (THI) questionnaire. The THI is widely used to assess the impact of tinnitus on the quality of life [36]. It consists of 25 items, with responses scored as follows: “no” = 0 points, “sometimes” = 2 points, and “yes” = 4 points. The total score ranges from 0 to 100, with higher scores indicating a greater distress due to tinnitus. The items are categorized into three subscales: functional (11 items), emotional (9 items), and catastrophic (5 items). Based on the total score, the severity of tinnitus is classified as follows: “slight” (0–16), “mild” (18–36), “moderate” (38–56), “severe” (58–76), and “catastrophic” (78 and above). In this study, participants with a score of 36 or below were classified into the mild group, and those with a score of 38 or above were classified into the severe group. We aimed to examine the differences in demographics, hearing, and personality traits between the two groups.

The Beck Depression Inventory (BDI) was used to assess individual levels of depression. Since its introduction by Beck et al. in 1961, the BDI has been the most widely used psychometric test for measuring the severity of depression [37]. It was revised to BDI-II in 1996 [38]. However, as the BDI-II version is a copyrighted inventory, the original version of the BDI, which is freely available for non-commercial use, is commonly used in various domains for screening depressive symptoms. In our study, we administered the Korean version of the original BDI (The Seoul Youth Clinic, Seoul, Republic of Korea). The questionnaire consists of 21 items, with each participant rating their feelings at the time of response on a scale from 0 to 3. The total score ranges from 0 to 63. Scores are classified as follows: 0–9 indicates “no depression”, 10–15 indicates “mild depression”, 16–23 indicates “moderate depression”, and 24 or above indicates “severe depression”.

### 2.4. Big Five Personality Test

The Big Five personality traits, also known as the “five-factor model of personality,” group five distinct characteristics used in personality research. Initially developed in the 1980s within the field of psychological trait theory, these traits were consolidated into the following five factors by the 1990s [39]: Openness, Conscientiousness, Extraversion, Agreeableness, and Neuroticism. The Big Five personality traits are widely used as a personality assessment tool globally. Various versions of the Big Five personality trait tests have emerged, each with slight modifications and improvements since the 2000s.

In this study, we used the Big Five Inventory test provided by Korea Guidance (Seongnam, Republic of Korea), a specialized psychological assessment company. This test is based on the Big Five Inventory proposed by John et al., encompassing the five factors and 30 facets [40]. In addition to the five factors of Openness, Conscientiousness, Extraversion, Agreeableness, and Neuroticism, this test uniquely categorizes certain facets into “ego-related” and “communication” domains. Therefore, it can be considered to comprise 7 factors and 25 facets. The ego-related domain includes the facets Ego Strength, Helplessness, Emotional Trauma, and Person Dependency, while the communication domain includes the facets Listening and Expression.

Participants completed this test online through the website. The results were provided as T-scores and percentile scores. T-scores are standardized scores with a mean of 50 and a standard deviation of 10, allowing comparison with a nationwide sample of individuals in similar age groups. This method adjusts for age-related differences, providing a distinct advantage. Percentile scores represent cumulative percentages, indicating where a participant’s score ranks among 100 individuals. Essentially, T-scores and percentile scores convey the same information, with the latter being more intuitive on a 100-point score. For this study, we used the more intuitive percentile scores on a 100-point scale.

### 2.5. Statistical Analyses

Comparisons of continuous variables between groups were performed using Student’s *t*-test, while categorical variables were compared using the chi-square test. For multivariate analysis, a multiple linear regression model was constructed. Initially, simple linear regression was conducted for each variable, and those with significance were included in the multiple linear regression model. The inclusion of these variables in the final model was determined through the stepwise method. All statistical analyses were performed using IBM SPSS Statistics for Windows (ver. 23.0; IBM Corp., Armonk, NY, USA). A significance level of *p* < 0.05 was used to determine statistical significance.

## 3. Results

### 3.1. Demographics and Hearing Levels of Subjects

Out of the 32 participants, 15 had a THI score of 36 or below and were classified into the mild group, while the remaining 17 were classified into the severe group. There was no significant age difference between the groups; however, the mild group had a higher proportion of males (86.7%), whereas the severe group had a higher proportion of females (64.7%) (*p* = 0.010). As expected, the severe group had significantly higher THI scores across all domains. Although individuals diagnosed with depression and taking medication were excluded from the study, the overall BDI scores were relatively low, yet the severe group had significantly higher BDI scores (9.7 ± 7.3) compared to the mild group (4.9 ± 2.5) (*p* = 0.018). Pure-tone thresholds at 500 Hz and 1000 Hz were significantly higher in the severe group. There was no difference in speech discrimination between the two groups (Table 1).

### 3.2. Differences in the Five Factors

**Figure 1:**
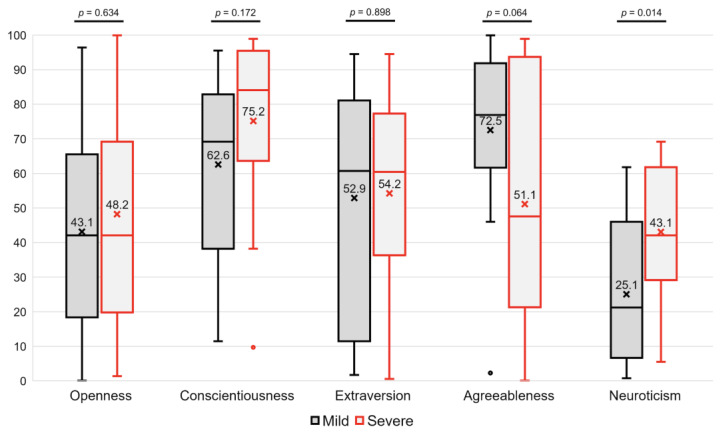
Differences in the Big Five Inventory between two groups.

The differences in the percentile scores for Openness, Conscientiousness, Extraversion, Agreeableness, and Neuroticism between the two groups are presented in Figure 1. In the Openness domain, the mild group had a percentile score of 43.1 ± 30.1, while the severe group scored 48.2 ± 29.6, with no significant difference between them (*p* = 0.634). For Conscientiousness, the severe group scored slightly higher at 75.2 ± 25.9 compared to the mild group at 62.6 ± 24.8, but this difference was not statistically significant (*p* = 0.172). Similarly, there were no significant differences in the Extraversion (52.9 ± 32.5 in the mild group and 54.2 ± 27.2 in the severe group, *p* = 0.898) and Agreeableness domains (72.5 ± 24.8 and 51.1 ± 36.2, respectively, *p* = 0.064). However, in the Neuroticism domain, the severe group scored significantly higher at 43.0 ± 18.2 compared to 25.1 ± 21.0 in the mild group (*p* = 0.014) (Figure 1).

### 3.3. Differences in the Ego- and Communication-Related Factors

Among the ego-related factors, there was no significant difference in Ego Strength between the two groups (65.0 ± 29.2 in the mild group and 62.0 ± 29.5 in the severe group, *p* = 0.778). The most pronounced difference between the two groups was observed in the Helplessness domain. Participants in the mild group had a score of 26.3 ± 22.9, whereas those in the severe group had a score of 62.3 ± 27.9 (*p* < 0.001). In the Emotional Trauma domain, the mild group scored 24.5 ± 22.3 and the severe group scored 36.7 ± 31.0. Although the severe group had higher scores, the difference was not statistically significant in this simple comparison (*p* = 0.217). There was little difference in Person Dependency between the groups (36.2 ± 19.7 and 31.4 ± 27.1, respectively, *p* = 0.571).

**Figure 2:**
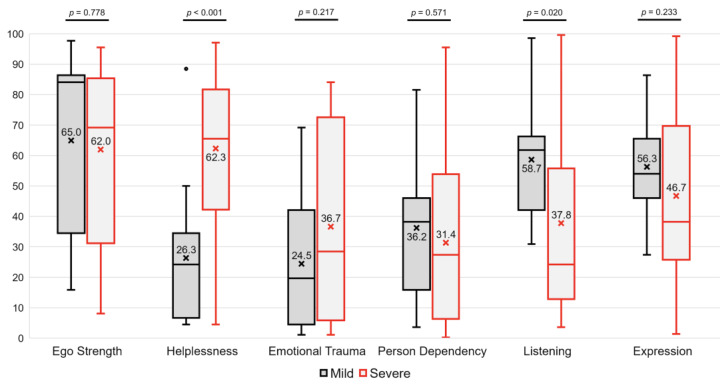
Differences in the ego-related (Ego Strength, Helplessness, Emotional Trauma, and Person Dependency) and the communication-related (Listening and Expression) factors between two groups.

In the communication-related factors, there was a significant difference between the groups in the Listening domain. Subjects in the mild group had a score of 58.7 ± 18.8, which was higher than the score of 37.8 ± 27.9 in the severe group (*p* = 0.020). There was no significant difference in the Expression (56.3 ± 15.6 in the mild group and 46.7 ± 27.8 in the severe group, *p* = 0.233) (Figure 2).

### 3.4. A Multivariate Analysis to Identify Factors Affecting the THI Score

Simple group comparisons alone have limitations in determining the extent to which each factor influences the distress of tinnitus. Therefore, to identify the factors that independently affect the tinnitus distress, we created a multiple linear regression model. First, we performed simple linear regression for each variable to identify significant ones. The variables found to have a notable correlation with the THI score were ‘Gender (female)’, ‘Agreeableness’, ‘Neuroticism’, ‘Helplessness’, ‘Emotional Trauma’, ‘Listening’, ‘Expression’, ‘BDI’, ‘Pure-tone threshold at 500 Hz’, ‘Pure-tone threshold at 1000 Hz’, ‘Pure-tone threshold at 2000 Hz’, and ‘Pure-tone threshold at 4000 Hz’ (Table 2).

Using these variables, we constructed a multiple linear regression model. Variables were selected for inclusion in the model using the stepwise method. Ultimately, the model included ‘Helplessness’ (estimate 0.419; 95% confidence interval; 0.249 to 0.589; *p* < 0.001), ‘Emotional Trauma’ (0.213; 0.020 to 0.406; *p* = 0.032), and ‘Pure-tone threshold at 2000 Hz’ (0.944; 0.477 to 1.411; *p* < 0.001). The model using three variables demonstrated a relatively high explanatory power with an R^2^ of 0.637 (Table 3).

## 4. Discussion

In this study, subjects in the severe group had significantly higher Neuroticism scores. This finding is consistent with several previous studies. For example, Karaaslan et al. compared patients with tinnitus to a control group without tinnitus and found an association between tinnitus and the Neuroticism trait [22]. Similarly, other studies examining differences based on tinnitus severity, rather than comparing to a healthy control group, have also found a relationship with Neuroticism [27]. Comprehensive scoping or systematic review studies also highlight the association between the Neuroticism and tinnitus [21,41]. Consequently, it has been suggested that patients with high levels of Neuroticism may benefit from a more personalized treatment approach compared to those with general chronic subjective tinnitus [24].

However, in our study, when analyzing the relationship between personality traits and tinnitus severity based on the correlation with THI scores, Neuroticism was not included as a significant factor. Interestingly, while studies conducted primarily in Western cultures consistently show an association between Neuroticism and tinnitus, studies from East Asian regions, though limited, do not consistently demonstrate this association. A study from China using the Eysenck Personality Questionnaire found that Neuroticism scores were not significantly higher in patients with tinnitus [32]. In a study conducted on Koreans, the Temperament and Character Inventory (TCI) was used, making direct comparisons with other studies based on the Big Five modality somewhat difficult; however, it was found that tinnitus patients had higher ‘Harm avoidance’ scores [33]. ‘Harm avoidance’ is characterized by excessive worry, doubt, and fatigue, and has been regarded as a potential factor in the development of neurotic disorders, making it similar to the Neuroticism domain [42]. This study also found that while tinnitus patients had higher scores, there was no significant correlation between ‘Harm avoidance’ scores and tinnitus severity. This pattern is quite similar to our findings. In summary, combining the results of our study with the two existing studies suggests that the personality types of tinnitus patients may present differently in East Asian populations. Therefore, it is clear that more studies need to be conducted in Asian populations to accumulate data and further explore the association.

The multivariate analysis revealed significant results for Helplessness and Emotional Trauma facets. Including the pure-tone threshold at 2 kHz in the model resulted in an R^2^ value of 0.637, indicating a model with relatively high explanatory power. Ultimately, our researchers also believe that Helplessness and Emotional Trauma are more critical factors than Neuroticism. Therefore, we would like to discuss these findings further. The association between tinnitus and posttraumatic stress disorder (PTSD) has been reported previously. A study by Moring et al. similarly divided subjects into mild and severe tinnitus groups and found that the PTSD total severity was higher in the severe tinnitus group [43]. Some perspectives suggest that traumatic memory and tinnitus share mechanisms in their pathophysiology [44]. While it is questionable whether the emotional stress experienced by our study subjects would be substantial enough to be diagnosed as PTSD, it is evident that addressing emotional stress should be part of the management and counseling process for tinnitus patients.

Individuals diagnosed with depression were not included in the study, and even though the average BDI score of the severe group was only 9.7, which is on the borderline between no depression and mild depression, the fact that the severe group showed significantly more Helplessness compared to the mild group is quite an intriguing finding. Furthermore, the multivariate analysis examining the relationship with THI also revealed a significant correlation. Helplessness is considered to be associated with clinical depression, particularly in relation to the well-known theory of ‘learned helplessness’, which suggests that persistent learned helplessness can lead to depression [45]. However, it is challenging to determine from this study whether Helplessness is a factor that exacerbates tinnitus (i.e., Helplessness as a precursor to tinnitus) or whether it is induced by the distress caused by tinnitus (i.e., tinnitus as a precursor to Helplessness). Nevertheless, it seems clear that addressing the Helplessness experienced by tinnitus patients should be an integral part of the treatment process. Both the aspects related to Emotional Trauma and Helplessness can be addressed through counseling between the clinician and the patient. These factors might explain why therapies like TRT and CBT have been shown to be effective in tinnitus treatment, as supported by previous research findings [46,47].

The finding that a higher pure-tone threshold at 2000 Hz (indicating worse hearing) correlates with greater distress from tinnitus aligns with clinical guidelines that recommend hearing aids for those with hearing loss [17,19,20,48]. It is important to note, however, that our study did not include patients with ≥ moderate hearing loss. This ultimately pertains to whether the use of hearing aids is beneficial for mild hearing loss. Our results support existing research suggesting that hearing aids may be recommended for individuals with tinnitus, even with mild hearing loss [49,50,51,52]. Together with the personality factors discussed earlier, this is also an independent factor. Therefore, even with mild hearing loss, if the distress from tinnitus is significant, the use of hearing aids should be considered. While personality factors may be beyond our ability to alter, improving hearing through the use of hearing aids is an achievable intervention, making their use significant in managing tinnitus.

It is necessary to investigate the future course of tinnitus in the participants of this study. According to research by Simões et al., individuals with lower Neuroticism scores and higher Extraversion scores clinically show greater improvement in tinnitus symptoms [23]. Additionally, a study by Kleinstäuber et al. found that individuals with higher scores in the Openness domain had better treatment outcomes, attributed to their greater acceptance of CBT [26]. Our researchers believe that there is a need to develop a shortened, modified personality test specifically for tinnitus patients. To achieve this, it is essential to identify the questions in personality tests that are associated with tinnitus. By identifying items related not only to the severity of tinnitus but also to factors influencing its progression, we can develop a new personality test tailored for tinnitus patients. The subjects included in this study are currently being followed up to monitor their treatment processes, which is expected to provide insights into the impact of personality on tinnitus.

This study has some limitations. Although we obtained several statistically significant results, the small number of participants makes it difficult to generalize these findings. Additionally, as the study compares differences between the two groups in a cross-sectional manner without examining the prognosis of tinnitus in these individuals, its applicability in clinical setting is limited. This study was designed as a preliminary investigation. Considering these limitations, future research should aim to recruit more participants and compare treatment prognoses to better understand how personality trait analysis might be beneficial in clinical practice.

## 5. Conclusions

Participants belonging to the group with greater tinnitus distress had higher scores in the Neuroticism domain of the Big Five Inventory. Through multivariate analyses, the factors influencing tinnitus severity were identified as Helplessness, Emotional Trauma, and the pure-tone threshold at 2000 Hz.

## Figures and Tables

**Table 1:** Demographics of subjects (N = 32).

	Mild (n = 15)	Severe (n = 17)	*p*-Value
Age	47.9 ± 12.2	50.2 ± 7.4	0.515
Gender			0.010
Female	2 (13.3%)	11 (64.7%)	
Male	13 (86.7%)	6 (35.3%)	
Tinnitus Handicap Inventory (THI)			
Functional score	10.8 ± 5.2	25.2 ± 6.4	<0.001
Emotional score	7.3 ± 4.3	24.8 ± 6.6	<0.001
Catastrophic score	6.4 ± 3.6	12.9 ± 4.1	<0.001
Total score	24.5 ± 8.6	62.9 ± 12.8	<0.001
Beck Depression Inventory (BDI)	4.9 ± 2.5	9.7 ± 7.3	0.018
Pure-tone threshold (dB HL)			
500 Hz	11.2 ± 5.8	17.9 ± 8.4	0.014
1000 Hz	11.5 ± 7.4	19.7 ± 11.6	0.026
2000 Hz	13.7 ± 9.6	20.6 ± 12.2	0.088
4000 Hz	25.0 ± 17.2	32.2 ± 16.1	0.230
8000 Hz	33.5 ± 24.0	37.2 ± 21.6	0.649
Speech discrimination (%)	96.8 ± 2.4	95.9 ± 4.9	0.501

**Table 2:** Factors affecting the score of the Tinnitus Handicap Inventory (univariate analyses).

Variables	Estimate	95% Confidence Interval	*p*-Value
Age	0.275	−0.566 to 1.117	0.509
Gender (female)	19.917	5.007 to 34.847	0.011
Openness	0.069	−0.212 to 0.350	0.619
Conscientiousness	0.184	−0.131 to 0.498	0.243
Extraversion	−0.047	−0.330 to 0.236	0.738
Agreeableness	−0.279	−0.510 to −0.047	0.020
Neuroticism	0.377	.0012 to 0.742	0.043
Ego Strength	0.003	−0.285 to 0.290	0.984
Helplessness	0.387	0.163 to 0.612	0.001
Emotional Trauma	0.322	0.045 to 0.599	0.024
Person Dependency	−0.154	−0.501 to 0.192	0.371
Listening	−0.286	−0.588 to 0.017	0.063
Expression	−0.296	−0.639 to 0.048	0.087
Beck Depression Inventory	1.755	0.543 to 2.967	0.006
Pure-tone threshold at 500 Hz	1.436	0.543 to 2.328	0.003
Pure-tone threshold at 1000 Hz	1.007	0.316 to 1.698	0.006
Pure-tone threshold at 2000 Hz	0.867	0.216 to 1.517	0.011
Pure-tone threshold at 4000 Hz	0.452	−0.015 to 0.919	0.057
Pure-tone threshold at 8000 Hz	0.139	−0.228 to 0.506	0.444
Speech discrimination	−0.891	−2.995 to 1.212	0.394

**Table 3:** Factors affecting the score of the Tinnitus Handicap Inventory (a multivariate analysis; R^2^ = 0.637).

Variables ^1^	Estimate	95% Confidence Interval	*p*-Value
Helplessness	0.419	0.249 to 0.589	<.0001
Emotional Trauma	0.213	0.020 to 0.406	0.032
Pure-tone threshold at 2000 Hz	0.944	0.477 to 1.411	<0.001

^1^ *p* values ≤ 0.200 in univariate analyses were included in the multivariate analysis (multiple linear regression). ‘Gender (Female)’, ‘Agreeableness’, ‘Neuroticism’, ‘Listening’, ‘Expression’, ‘Beck Depression Inventory’, ‘PTA threshold at 500 Hz’, ‘PTA threshold at 1000 Hz’, and ‘PTA threshold at 4000 Hz’ were initially included as variable for the multivariate analysis but excluded by the stepwise method.

## Data Availability

The data presented in this study are available on request from the corresponding author.
